# Author Correction: Paternal grandfather’s access to food predicts all-cause and cancer mortality in grandsons

**DOI:** 10.1038/s41467-021-22367-x

**Published:** 2021-03-23

**Authors:** Denny Vågerö, Pia R. Pinger, Vanda Aronsson, Gerard J. van den Berg

**Affiliations:** 1 CHESS, Centre for Health Equity Studies, Department of Public Health Sciences, SE-106 91 Stockholm University, Stockholm, Sweden; 2 Department of Economics, University of Bonn, Adenauerallee 24-42, 53113 Bonn, Germany; 3 briq, Institute on Behavior & Inequality, Bonn, Germany; 4 Department of Economics, Priory Rd Complex, University of Bristol, Bristol, BS8 ITU UK; 5 IFAU, Institute for Evaluation of Labor Market and Education Policy, Uppsala, Sweden

**Keywords:** Nutrition, Epidemiology, Risk factors, Social sciences

Correction to: *Nature Communications* 10.1038/s41467-018-07617-9, published online 11 December 2018

The original version of the Supplementary information associated with this Article contained an error in Supplementary Table 7 in which the power calculations were incorrect as a result of an error in the standard deviation of the exposure variable when performing the calculations. The correct version of Supplementary Table 7 is:

Supplementary Table 7: What is the power in our replication to detect the most important results in the Överkalix studies?

**Table:** 

**All-cause mortality result (Kaati et al., 2007)**						
***Males***						
*Food access*	*Exposed ancestor*	*Hazard Ratio*	*p*	*Deaths_Överkalix*	*Deaths/Total N_replication*	*POWER*
good	father	1.70	0.01	146	3419/3820	**>0.99**
good	paternal grandfather	1.45	0.05	164	339/3224	**0.48**
poor	paternal grandfather	0.60	0.01	164	339/3224	**0.62**
***Females***						
*Food access*	*Exposed ancestor*	*Hazard Ratio*	*p*	*Deaths_ Överkalix*	*Deaths/Total N, replication*	*POWER*
good	paternal grandmother	1.75	0.01	139	222/3051	**0.50**
poor	paternal grandmother	0.71	0.01	135	222/3051	**0.30**
**Diabetes and cardiovascular mortality results (Kaati et al., 2002)**						
***Diabetes***, ***males and females combined***						
*Food access*	*Exposed ancestor*	*Odds Ratio*	*p*	*Deaths/Total N_ Överkalix*	*Deaths*/*Total N*_ *replication*	*POWER*
good	father	0.14	0.06	19/239	544/7280	**>0.99**
good	paternal grandfather	2.34	0.09	19/239	26/6275	**0.49**
poor	paternal grandfather	0.35	0.09	19/239	26/6275	**0.06**
poor	maternal grandmother	2.73	0.06	19/239	41/5891	**0.54**
***CVD***, ***males and females combined***						
*Food access*	*Exposed ancestor*	*Odds Ratio*	*p*	*Deaths/Total N_ Överkalix*	*Deaths*/*Total N*_ *replication*	*POWER*
poor	father	0.42	0.05	128/239	3846/7280	**>0.99**

Notes: Hazard ratios and odds ratios as reported in Kaati et al. (2002) and Kaati et al. (2007). The power analyses for all-cause mortality (one-sided test) were computed using Schoenfeld’s sample size-formula for the proportional hazards regression model. For Diabetes and CVD mortality the power was computed using a two-sample proportions test (one-sided test, only decreased individuals in UBCoS were used to determine the sample size).

which replaces the previous incorrect version:

Supplementary Table 7: What is the power in our replication to detect the most important results in the Överkalix studies?

**Table:** 

**All-cause mortality results (Kaati et al., 2007)**						
***Males***						
*Food access*	*Exposed ancestor*	*Hazard Ratio*	*p*	*Deaths_Överkalix*	*Deaths/Total N_replication*	*POWER*
good	father	1.70	0.01	146	3419/3820	**>0.99**
good	paternal grandfather	1.45	0.05	164	339/3224	**0.93**
poor	paternal grandfather	0.60	0.01	164	339/3224	**0.99**
***Females***						
*Food access*	*Exposed ancestor*	*Hazard Ratio*	*p*	*Deaths_ Överkalix*	*Deaths/Total N, replication*	*POWER*
good	paternal grandmother	1.75	0.01	139	222/3051	**0.99**
poor	paternal grandmother	0.71	0.01	135	222/3051	**0.72**
**Diabetes and cardiovascular mortality results (Kaati et al., 2002)**						
***Diabetes***, ***males and females combined***						
*Food access*	*Exposed ancestor*	*Odds Ratio*	*p*	*Deaths/Total N_ Överkalix*	*Deaths*/*Total N*_ *replication*	*POWER*
good	father	0.14	0.06	19/239	544/7280	**>0.99**
good	paternal grandfather	2.34	0.09	19/239	26/6275	**0.76**
poor	paternal grandfather	0.35	0.09	19/239	26/6275	**0.87**
poor	maternal grandmother	2.73	0.06	19/239	41/5891	**0.90**
***CVD***, ***males and females combined***						
*Food access*	*Exposed ancestor*	*Odds Ratio*	*p*	*Deaths/Total N_ Överkalix*	*Deaths*/*Total N*_ *replication*	*POWER*
poor	father	0.42	0.05	128/239	3846/7280	**>0.99**

Notes: Relative probabilities were reconstructed from sample sizes and odds ratios reported in (Kaati et al., 2002). The power analyses for all-cause mortality were computed using Schoenfeld’s sample-size formula for the proportional-hazards regression model. For Diabetes and CVD mortality the power was computed using a two-sample proportions test (only deceased individuals in UBCoS were used to determine the sample size).

Furthermore, the original version of this Article contained an error in the second paragraph of the ‘Results’ section, which incorrectly read ‘In analyses of G2 mortality this power varied from 72 to 99%.’ The correct version reads ‘In analyses of G2 mortality this power was modest’. These have been corrected in both the PDF and HTML versions of the Article and Supplementary information file. The HTML has also been updated to include a corrected version of the Supplementary information.

## Supplementary information

Supplementary information

