# Supplementary material for: Paternal grandfather’s access to food predicts all-cause and cancer mortality in grandsons
Source: Nat Commun. 2018 Dec 11;9:5124. doi: 10.1038/s41467-018-07617-9 (PMC6290014; doi:10.1038/s41467-018-07617-9)
Supplement: Supplementary file 1 — Supplementary Information [file 41467_2018_7617_MOESM1_ESM.pdf]

Supplementary Information. Cover page

Vågerö et al. Paternal grandfather's access to food influences all-cause and cancer mortality in grandsons

This document consists of Supplementary Tables 1-7

Supplementary Table 1: All-cause mortality in G2 men and women by grandparental harvest exposures in SGP: hazard ratios with 95% confidence limits (in brackets) based on Cox regression. Mortality follow-up 1961-2015.

	**Men**				**Women**			
	Model 1		Model 2		Model 1		Model 2	
**Maternal grandmother**								
Good	0.84	[0.52,1.36]	0.89	[0.53,1.48]	1.05	[0.65,1.71]	1.01	[0.61,1.69]
Intermediate	1.00	ref	1.00	ref	1.00	ref	1.00	ref
Poor	0.92	[0.57,1.48]	0.95	[0.58,1.55]	0.52	[0.23,1.18]	0.51	[0.24,1.11]
**Maternal grandfather**								
Good	0.73	[0.43,1.25]	0.74	[0.44,1.24]	0.77	[0.41,1.46]	0.79	[0.40,1.56]
Intermediate	1.00	ref	1.00	ref	1.00	ref	1.00	ref
Poor	0.76	[0.46,1.27]	0.76	[0.49,1.19]	1.15	[0.62,2.11]	1.17	[0.64,2.15]
Observations	2987		2987		2904		2904	
Number of deaths	377		377		253		253	
	Model 1		Model 2		Model 1		Model 2	
**Paternal grandmother**								
Good	0.88	[0.46,1.66]	0.93	[0.49,1.76]	0.82	[0.38,1.75]	0.85	[0.40,1.81]
Intermediate	1.00	ref	1.00	ref	1.00	ref	1.00	ref
Poor	0.68	[0.36,1.28]	0.70	[0.35,1.37]	1.11	[0.60,2.06]	1.13	[0.62,2.08]
**Paternal grandfather**								
Good	1.50[a]	[0.99,2.26]	**1.55**[b]	[1.02,2.35]	0.71[a]	[0.36,1.40]	0.74[b]	[0.38,1.46]
Intermediate	1.00	ref	1.00	ref	1.00	ref	1.00	ref
Poor	0.92	[0.52,1.62]	0.93	[0.51,1.68]	1.08	[0.60,1.94]	1.11	[0.63,1.94]
Observations	3224		3224		3051		3051	
Number of deaths	339		339		222		222	

Statistically significant estimates (95% CI) in bold type

Model 1: Adjusted for G2 birth year, sibship size and sibling order, mother's/father's harvest exposure in SGP, social class, income and education, and any parental death before age 18

Model 2: + linear trends for grandparents birth years, with confidence limits based on sibling cluster robust standard errors

[a] Interaction: $p = 0.065$

[b] Interaction: $p = 0.053$

Supplementary Table 2: CVD and diabetes mortality in G2 by grandparental harvest exposures in SGP: hazard ratios with 95% confidence limits (in brackets) based on Cox regression. Mortality follow-up 1961-2015.

	**CVD mortality**			
	Model 1		Model 2	
**Maternal grandmother**				
Good	0.81	[0.44,1.50]	0.90	[0.50,1.63]
Intermediate	1.00	ref	1.00	ref
Poor	0.69	[0.32,1.48]	0.73	[0.36,1.48]
**Maternal grandfather**				
Good	0.93	[0.48,1.83]	0.97	[0.46,2.04]
Intermediate	1.00	ref	1.00	ref
Poor	0.82	[0.40,1.67]	0.83	[0.44,1.59]
Observations	5891		5891	
Number of deaths	204		204	
	Model 1		Model 2	
**Paternal grandmother**				
Good	0.77	[0.28,2.09]	0.74	[0.27,2.03]
Intermediate	1.00	ref	1.00	ref
Poor	1.07	[0.52,2.20]	1.05	[0.49,2.25]
**Paternal grandfather**				
Good	1.05	[0.53,2.06]	0.99	[0.50,1.95]
Intermediate	1.00	ref	1.00	ref
Poor	0.84	[0.39,1.81]	0.81	[0.39,1.69]
Observations	6275		6275	
Number of deaths	176		176	
	**Diabetes mortality**			
	Model 1		Model 2	
**Maternal grandmother**				
Good	2.25	[0.86,5.88]	**3.38**	[1.18,9.65]
Intermediate	1.00	ref	1.00	ref
Poor	0.96	[0.22,4.14]	1.12	[0.31,4.07]
**Maternal grandfather**				
Good	0.46	[0.06,3.42]	0.55	[0.07,4.61]
Intermediate	1.00	ref	1.00	ref
Poor	0.52	[0.07,3.79]	0.59	[0.08,4.26]
Observations	5891		5891	
Number of deaths	41		41	
	Model 1		Model 2	
**Paternal grandmother**				
Good	1.24	[0.16,9.34]	1.25	[0.15,10.12]
Intermediate	1.00	ref	1.00	ref
Poor	0.90	[0.12,6.87]	0.91	[0.12,6.69]
**Paternal grandfather**				
Good	0.00	[0.00,]	**0.00**	[0.00,]
Intermediate	1.00	ref	1.00	ref
Poor	1.95	[0.45,8.43]	1.92	[0.40,9.32]
Observations	6275		6275	
Number of deaths	26		26	

Statistically significant estimates (95% CI) in bold type

Model 1: Adjusted for G2 gender, birth year, sibship size and sibling order, mother's/father's harvest exposure in SGP, social class, income and education, and any parental death before age 18

Model 2: + linear trends for grandparents birth years, with confidence limits based on sibling cluster robust standard errors

Supplementary Table 3: Cancer mortality in G2 men and women by grandparental harvest exposures in SGP: hazard ratios with 95% confidence limits (in brackets) based on Cox regression. Mortality follow-up 1961-2015.

	**Men**				**Women**			
	**All cancers**							
	Model 1		Model 2		Model 1		Model 2	
**Maternal grandmother**								
Good	0.93	[0.41,2.14]	0.89	[0.33,2.39]	0.66	[0.29,1.51]	0.63	[0.27,1.48]
Intermediate	1.00	ref	1.00	ref	1.00	ref	1.00	ref
Poor	1.36	[0.65,2.84]	1.32	[0.63,2.76]	0.36	[0.09,1.46]	0.35	[0.09,1.39]
**Maternal grandfather**								
Good	0.45	[0.14,1.44]	0.43	[0.13,1.40]	0.62	[0.23,1.69]	0.66	[0.25,1.78]
Intermediate	1.00	ref	1.00	ref	1.00	ref	1.00	ref
Poor	0.54	[0.20,1.49]	0.53	[0.20,1.41]	1.45	[0.67,3.14]	1.51	[0.70,3.29]
Observations	2987		2987		2904		2904	
Number of deaths	124		124		129		129	
	Model 1		Model 2		Model 1		Model 2	
**Paternal grandmother**								
Good	1.09	[0.39,3.00]	1.20	[0.40,3.62]	0.87	[0.32,2.39]	0.93	[0.35,2.47]
Intermediate	1.00	ref	1.00	ref	1.00	ref	1.00	ref
Poor	1.37	[0.59,3.18]	1.45	[0.63,3.34]	0.98	[0.40,2.41]	1.01	[0.42,2.46]
**Paternal grandfather**								
Good	**3.35**[a]	[1.95,5.76]	**3.44**[b]	[1.87,6.34]	0.73[a]	[0.30,1.80]	0.74[b]	[0.29,1.89]
Intermediate	1.00	ref	1.00	ref	1.00	ref	1.00	ref
Poor	0.65	[0.20,2.06]	0.63	[0.20,1.99]	0.66	[0.24,1.81]	0.67	[0.25,1.79]
Observations	3224		3224		3051		3051	
Number of deaths	117		117		119		119	
	**Cancers not related to smoking**							
	Model 1		Model 2		Model 1		Model 2	
**Paternal grandmother**								
Good	0.00	[0.00,]	**0.00**	[0.00,]	0.93	[0.29,2.99]	1.03	[0.32,3.35]
Intermediate	1.00	ref	1.00	ref	1.00	ref	1.00	ref
Poor	1.01	[0.31,3.28]	1.13	[0.35,3.65]	0.83	[0.26,2.66]	0.88	[0.28,2.77]
**Paternal grandfather**								
Good	**3.51**[c]	[1.77,6.97]	**4.39**[d]	[2.02,9.53]	0.58[c]	[0.18,1.86]	0.59[d]	[0.18,1.96]
Intermediate	1.00	ref	1.00	ref	1.00	ref	1.00	ref
Poor	0.78	[0.19,3.26]	0.86	[0.21,3.52]	0.45	[0.11,1.85]	0.45	[0.11,1.85]
Observations	3224		3224		3051		3051	
Number of deaths	70		70		84		84	
	**Cancers related to smoking**							
	Model 1		Model 2		Model 1		Model 2	
**Paternal grandmother**								
Good	2.68	[0.91,7.84]	2.63	[0.77,9.02]	0.80	[0.11,5.92]	0.79	[0.12,5.30]
Intermediate	1.00	ref	1.00	ref	1.00	ref	1.00	ref
Poor	2.27	[0.67,7.73]	2.28	[0.70,7.41]	1.21	[0.28,5.27]	1.21	[0.32,4.57]
**Paternal grandfather**								
Good	**3.22**[e]	[1.33,7.81]	2.45[f]	[0.94,6.37]	1.15[e]	[0.27,4.86]	1.17[f]	[0.30,4.67]
Intermediate	1.00	ref	1.00	ref	1.00	ref	1.00	ref
Poor	0.48	[0.06,3.56]	0.40	[0.06,2.74]	1.14	[0.26,4.91]	1.15	[0.32,4.17]
Observations	3224		3224		3051		3051	
Number of deaths	47		47		35		35	

Statistically significant estimates (95% CI) in bold type

Model 1: Adjusted for G2 birth year, sibship size and sibling order, mother's/father's harvest exposure in SGP, social class, income and education, and any parental death before age 18

Model 2: + linear trends for grandparents birth years, with confidence limits based on sibling cluster robust standard errors

[a] Interaction: $p = 0.006$

[b] Interaction: $p = 0.005$

[c] Interaction: $p = 0.013$

[d] Interaction: $p = 0.009$

[e] Interaction: $p = 0.222$

[f] Interaction: $p = 0.240$

Supplementary Table 4: All-cause mortality, CVD mortality and diabetes mortality in G1 men and women by parental harvest exposures in SGP: hazard ratios with 95% confidence limits (in brackets) based on Cox regression. Mortality follow-up 1952-2015.

	**Men**				**Women**			
	**All-cause mortality**							
	Model 1		Model 2		Model 1		Model 2	
**Mother**								
Good	1.15	[0.97,1.35]	1.14	[0.96,1.36]	1.02	[0.86,1.21]	1.01	[0.83,1.24]
Intermediate	1.00	ref	1.00	ref	1.00	ref	1.00	ref
Poor	1.04	[0.88,1.22]	1.03	[0.87,1.22]	0.95	[0.79,1.14]	0.95	[0.78,1.15]
**Father**								
Good	1.07	[0.93,1.24]	1.08	[0.93,1.26]	1.05	[0.89,1.24]	1.08	[0.91,1.27]
Intermediate	1.00	ref	1.00	ref	1.00	ref	1.00	ref
Poor	1.11	[0.95,1.30]	1.11	[0.96,1.30]	0.89	[0.74,1.07]	0.90	[0.73,1.10]
Observations	3820		3820		3460		3460	
Number of deaths	3419		3419		2758		2758	
	**CVD mortality**							
	Model 1		Model 2		Model 1		Model 2	
**Mother**								
Good	1.21	[0.99,1.48]	1.22	[0.99,1.49]	1.04	[0.83,1.30]	1.02	[0.80,1.31]
Intermediate	1.00	ref	1.00	ref	1.00	ref	1.00	ref
Poor	0.97	[0.79,1.20]	0.97	[0.78,1.21]	0.89	[0.70,1.13]	0.88	[0.71,1.10]
**Father**								
Good	1.04	[0.86,1.26]	1.07	[0.88,1.29]	1.09	[0.88,1.36]	1.10	[0.88,1.36]
Intermediate	1.00	ref	1.00	ref	1.00	ref	1.00	ref
Poor	1.10	[0.90,1.34]	1.11	[0.91,1.35]	0.92	[0.73,1.16]	0.91	[0.70,1.17]
Observations	3820		3820		3460		3460	
Number of deaths	2179		2179		1667		1667	
	**Diabetes mortality**							
	Model 1		Model 2		Model 1		Model 2	
**Mother**								
Good	1.17	[0.68,2.02]	1.20	[0.70,2.05]	1.06	[0.60,1.87]	1.04	[0.58,1.88]
Intermediate	1.00	ref	1.00	ref	1.00	ref	1.00	ref
Poor	0.93	[0.52,1.66]	0.93	[0.51,1.68]	0.80	[0.42,1.51]	0.78	[0.41,1.49]
**Father**								
Good	**1.75**[a]	[1.16,2.64]	**1.84**[b]	[1.21,2.79]	0.94[a]	[0.53,1.66]	0.89[b]	[0.50,1.58]
Intermediate	1.00	ref	1.00	ref	1.00	ref	1.00	ref
Poor	1.48	[0.90,2.44]	1.52	[0.91,2.55]	0.77	[0.41,1.46]	0.76	[0.39,1.47]
Observations	3820		3820		3460		3460	
Number of deaths	289		289		255		255	

Statistically significant estimates (95% CI) in bold type

Model 1: Adjusted for G1 birth year and sibling position, and parents' social class and marital status at G1 birth

Model 2: + linear trends for parents birth years, with confidence limits based on sibling cluster robust standard errors

[a] Interaction: $p = 0.076$

[b] Interaction: $p = 0.078$

Supplementary Table 5: CVD hospitalization or mortality in G2 by grandparental harvest exposures in SGP: hazard ratios with 95% confidence limits (in brackets) based on Cox regression. Follow-up 1961-2015.

	Model 1		Model 2	
**Maternal grandmother**				
Good	0.95	[0.78,1.16]	0.94	[0.75,1.19]
Intermediate	1.00	ref	1.00	ref
Poor	1.09	[0.88,1.35]	1.09	[0.87,1.37]
**Maternal grandfather**				
Good	1.00	[0.80,1.24]	1.01	[0.80,1.26]
Intermediate	1.00	ref	1.00	ref
Poor	1.11	[0.90,1.37]	1.12	[0.89,1.39]
Observations	5891		5891	
Number of events	1756		1756	

	Model 1		Model 2	
**Paternal grandmother**				
Good	0.92	[0.69,1.23]	0.92	[0.68,1.24]
Intermediate	1.00	ref	1.00	ref
Poor	0.85	[0.65,1.10]	0.84	[0.65,1.10]
**Paternal grandfather**				
Good	0.96	[0.77,1.20]	0.95	[0.75,1.20]
Intermediate	1.00	ref	1.00	ref
Poor	0.92	[0.71,1.17]	0.91	[0.70,1.18]
Observations	6275		6275	
Number of events	1550		1550	

Statistically significant estimates (95% CI) in bold type

Model 1: Adjusted for G2 gender, birth year, sibship size and sibling order, mother's/father's harvest exposure in SGP, social class, income and education, and any parental death before age 18

Model 2: + linear trends for grandparents birth years, with confidence limits based on sibling cluster robust standard errors

Supplementary Table 6: Diabetes hospitalization or mortality in G2 by grandparental harvest exposures in SGP: hazard ratios with 95% confidence limits (in brackets) based on Cox regression. Follow-up 1961-2015.

	Model 1		Model 2	
**Maternal grandmother**				
Good	1.13	[0.76,1.69]	1.22	[0.78,1.91]
Intermediate	1.00	ref	1.00	ref
Poor	1.02	[0.64,1.63]	1.06	[0.62,1.82]
**Maternal grandfather**				
Good	**0.48**	[0.25,0.90]	0.53	[0.25,1.12]
Intermediate	1.00	ref	1.00	ref
Poor	0.75	[0.45,1.27]	0.80	[0.47,1.35]
Observations	5891		5891	
Number of events	385		385	
	Model 1		Model 2	
**Paternal grandmother**				
Good	0.99	[0.55,1.76]	1.01	[0.58,1.77]
Intermediate	1.00	ref	1.00	ref
Poor	0.87	[0.49,1.56]	0.88	[0.50,1.54]
**Paternal grandfather**				
Good	1.08	[0.68,1.72]	1.13	[0.72,1.79]
Intermediate	1.00	ref	1.00	ref
Poor	1.16	[0.71,1.91]	1.20	[0.77,1.87]
Observations	6275		6275	
Number of events	329		329	

Statistically significant estimates (95% CI) in bold type

Model 1: Adjusted for G2 gender, birth year, sibship size and sibling order, mother's/father's harvest exposure in SGP, social class, income and education, and any parental death before age 18

Model 2: + linear trends for grandparents birth years, with confidence limits based on sibling cluster robust standard errors

Supplementary Table 7: What is the power in our replication to detect the most important results in the Överkalix studies?

**All-cause mortality results (Kaati et al., 2007)**						
Males						
Food access	*Exposed ancestor*	*Hazard Ratio*	*p*	*Deaths_Överkalix*	*Deaths/Total N_replication*	*POWER*
good	father	1.70	0.01	146	3419/3820	>**0.99**
good	paternal grandfather	1.45	0.05	164	339/3224	**0.93**
poor	paternal grandfather	0.60	0.01	164	339/3224	**0.99**
Females						
Food access	*Exposed ancestor*	*Hazard Ratio*	*p*	*Deaths_ Överkalix*	*Deaths/Total N, replication*	*POWER*
good	paternal grandmother	1.75	0.01	139	222/3051	**0.99**
poor	paternal grandmother	0.71	0.01	135	222/3051	**0.72**
**Diabetes and cardiovascular mortality results (Kaati et al., 2002)**						
Diabetes, males and females combined						
Food access	*Exposed ancestor*	*Odds Ratio*	*p*	*Deaths/Total N_ Överkalix*	*Deaths/Total N_ replication*	*POWER*
good	father	0.14	0.06	19/239	544/7280	>**0.99**
good	paternal grandfather	2.34	0.09	19/239	26/6275	**0.76**
poor	paternal grandfather	0.35	0.09	19/239	26/6275	**0.87**
poor	maternal grandmother	2.73	0.06	19/239	41/5891	**0.90**
CVD, males and females combined						
Food access	*Exposed ancestor*	*Odds Ratio*	*p*	*Deaths/Total N_ Överkalix*	*Deaths/Total N_ replication*	*POWER*
poor	father	0.42	0.05	128/239	3846/7280	>**0.99**

Notes: Relative probabilities were reconstructed from sample sizes and odds ratios reported in (Kaati et al., 2002). The power analyses for all-cause mortality were computed using Schoenfeld's sample-size formula for the proportional-hazards regression model. For Diabetes and CVD mortality the power was computed using a two-sample proportions test (only deceased individuals in UBCoS were used to determine the sample size).
